# Vitamin E Modifies High-Fat Diet-Induced Increase of DNA Strand Breaks, and Changes in Expression and DNA Methylation of *Dnmt1* and *MLH1* in C57BL/6J Male Mice

**DOI:** 10.3390/nu9060607

**Published:** 2017-06-14

**Authors:** Marlene Remely, Franziska Ferk, Sonja Sterneder, Tahereh Setayesh, Tatjana Kepcija, Sylvia Roth, Rahil Noorizadeh, Martina Greunz, Irene Rebhan, Karl-Heinz Wagner, Siegfried Knasmüller, Alexander Haslberger

**Affiliations:** 1 Department of Nutritional Sciences, University Vienna, 1010 Vienna, Austria; sonja.sterneder@gmail.com (S.S.); tatjana.kepcija@gmail.com (T.K.); rothsylvia@gmail.com (S.R.); martina.greunz@gmx.at (M.G.); irene.rebhan@gmx.at (I.R.); karl-heinz.wagner@univie.ac.at (K.-H.W.); alexander.haslberger@univie.ac.at (A.H.); 2 Institute of Cancer Research, Department of Medicine I, Medical University of Vienna, 1090 Vienna, Austria; franziska.ferk@meduniwien.ac.at (F.F.); sar.setayesh@yahoo.com (T.S.); a0922767@unet.univie.ac.at (R.N.); siegfried.knasmueller@meduniwien.ac.at (S.K.)

**Keywords:** *MLH1*, *Dnmt1*, DNA damage, gene expression, DNA methylation, SCGE assay

## Abstract

Obesity is associated with low-grade inflammation, increased ROS production and DNA damage. Supplementation with antioxidants might ameliorate DNA damage and support epigenetic regulation of DNA repair. C57BL/6J male mice were fed a high-fat (HFD) or a control diet (CD) with and without vitamin E supplementation (4.5 mg/kg body weight (b.w.)) for four months. DNA damage, DNA promoter methylation and gene expression of *Dnmt1* and a DNA repair gene (*MLH1*) were assayed in liver and colon. The HFD resulted in organ specific changes in DNA damage, the epigenetically important *Dnmt1* gene, and the DNA repair gene *MLH1*. Vitamin E reduced DNA damage and showed organ-specific effects on *MLH1* and *Dnmt1* gene expression and methylation. These results suggest that interventions with antioxidants and epigenetic active food ingredients should be developed as an effective prevention for obesity—and oxidative stress—induced health risks.

## 1. Introduction

Obesity is associated with a positive energy balance, an abnormal increase of adipose tissue and weight gain that impairs health [1]. Genetic factors such as single nucleotide polymorphisms, the environment, social status, dietary behavior, metabolism, microbiota, and physical activity are proposed to influence its development [2]. However, the adipose tissue is not merely an energy depot: it is also a highly active metabolic and endocrine organ. Various bioactive peptides, called adipokines, are involved in energy homeostasis, lipid and glucose metabolism, inflammation, fibrinolysis, coagulation, and blood pressure. Among others, they include cytokines such as interleukin-6 (*IL-6*) and tumor necrosis factor α (TNFα), leptin, monocyte chemoattractant protein-1 (MCP-1), plasminogen activator inhibitor-1 (PAI-1), adiponectin, and resistin [3,4].

In addition to adipocytokines and free fatty acids (FFAs), an increase of reactive oxygen species (ROS) production and increased oxidative stress are reported [5,6]. In obese mice, the ROS production increases in adipose tissue along with an elevated expression of nicotinamide adenine dinucleotide phosphate (NADPH) oxidase and decreased expression of antioxidative enzymes such as superoxide dismutase (SOD), glutathione peroxidase (GPX), and catalase (CAT) as well as altered production of adipocytokines in accumulated fat. Together with a positive correlation of biomarkers for systemic oxidative stress, it is likely that adipose tissue is the main source of elevated plasma ROS [7]. Oxidative stress is involved in both genome-wide hypomethylation and promoter hypermethylation of the DNA [8].

Increased levels of oxygen radicals are involved in DNA damage including base modifications, deletions, strand breaks, and chromosomal rearrangements, which interfere with DNA methylation [9,10]. Guanine within CpG dinucleotides is the favored base for oxidative damage, resulting in the production of 8-oxoguanine (8-oxoG). Substitution of guanine to 8-oxoG reduces the binding capacity of transcription repressor proteins causing persistent transcription of affected genes. However, the methyl group of 5-methylcytosine is similarly accessible to oxidation, forming 5-hydroxy-methylcytosine [10]. Another product of oxidative damage is 8-hydroxy-2-deoxyguanosine within CpG dinucleotides, leading to strong inhibition of cytosine methylation [9]. On the other hand, ROS-induced oxidative stress contributes to hypermethylation of normally unmethylated promoter regions, resulting in transcriptional silencing of key antioxidant enzymes as well as tumor suppressor genes [11].

O^6^-methylguanine-deoxyribonucleic acidmethyltransferase (MGMT) is a DNA repair enzyme, which is able to repair O^6^-methylguanine by eliminating mutagenic and cytotoxic alkyl groups [12,13]. Either the repair protein MutL homolog 1 (*MLH1*) is part of the DNA mismatch repair (MMR) system [14,15]. This correction of replication errors involves recognition of mismatches and selective removal of the affected DNA region. If the repair of DNA lesions is not accurate, an increase of DNA mutations is the consequence and can cause cellular dysfunctions and diseases including sporadic and hereditary human cancers. In patients with hereditary non-polyposis colorectal cancer, as well as a wide variety of other cancers, increased mutations in microsatellite sequences, known as microsatellite instability (MSI), are associated with defects in MMR system [16]. MSI is often associated with promoter hypermethylation, resulting in inactivation of *MLH1* [15,16].

The methyl donor *S*-Adenosylmethionin (SAM) and the expression of *DNA methyltransferase 1* (*Dnmt1*) are also impaired due to oxidative stress [17] and may promote health disorders: for example, in cancer, an increased expression has been mentioned [18]. In a mouse model of asthma disease *Dnmt1* was down-regulated [19]. Hodge et al. (2001, 2005, 2007) extensively studied the connection between *Dnmt1* and the inflammatory cytokine: IL-6. Treatment of cells with IL-6 increased the expression and the activity of *Dnmt1* due to transcriptional activation in the promoter [20,21,22]. Furthermore, the elevated expression of *Dnmt1* coordinated by IL-6 is negatively correlated with the expression level of tumor suppressor gene p53. It is suggested that *IL-6* has the ability to induce *p53* promoter methylation through up-regulation of *Dnmt1* [21]. Thus, chronic exposure to low-grade inflammation, especially IL-6, might induce dysregulation of the *Dnmt1* gene.

Nutritional interventions, e.g., with antioxidants, may improve DNA methylation and nucleotide biosynthesis reactions and as a consequence DNA repair. We reported previously that a diet rich in antioxidants (e.g., EGCG) [23] and vitamins (in particular folate) alters DNA methylation and ameliorates ROS induced epigenetic lesions [24]. It was shown that vitamin E plays a key role in antagonizing oxidative stress [25] as a direct scavenger of toxic free radicals, induction of antioxidant enzymes, enhancing inflammatory/immune response, modulation of DNA repair systems, and of signal transduction pathways [26]. In the colon, tocopherols react with lipid soluble peroxyl radicals and quench the further propagation of free radicals [27]. In the liver, oxidative stress is involved in the pathogenesis of various diseases such as non-alcoholic fatty liver disease (NAFLD). NAFLD is the hepatic manifestation of metabolic syndrome and frequently associated with diabetes, hyperlipidemia, and obesity. Antioxidant treatment of NAFLD with vitamin E is a well-established pharmacological approach [28].

Several earlier findings with rodent and humans indicate that obesity induces low-grade inflammatory processes in the following genomic instability [29,30,31]. One of the strategies to prevent adverse effects of obesity and co-morbidities may be nutritional interventions. In the present study, we investigated the impact of vitamin E intake on genomic instability, DNA methylation, and gene expression of *Dnmt1*, and of the DNA repair molecule *MLH1*, assayed in liver and colon of C57BL/6J mice. The colon plays an important role in nutrient absorption, while the liver has important impact on glucose and insulin metabolism as the main organ for insulin clearance from blood. DNA damage was measured in single cell gel electrophoresis (SCGE) experiments which are based on the determination of DNA migration in an electric field: the extent of the size of “comets” reflects formation of single and double strand breaks and apurinic site [32]. DNA methylation was analyzed with bisulfite converted DNA in a Pyromark, and the expression of candidate genes was assayed from reverse-transcribed complementary DNA (cDNA).

## 2. Materials and Methods

The animal experiment was approved by the Ethical Committee of the Medical University of Vienna (BMWFW-66.009/0329-WF/V/3b/2014). Six-week-old C57BL/6J male mice (*n* = 60, 15 mice/group, Janvier Labs, France) were used for the animal experiment. Three animals were kept per cage (Macrolon type III, Techniplast GmbH, Hohenpeißenberg, Germany) under standard conditions (24 ± 1 °C, humidity 50% ± 5%, 12 h light/dark cycle); food and water were provided ad libitum. After 14 days of acclimatization with CD (control diet, EF R/M Control, 11 kJ % fat, ssniff Spezialdiäten GmbH, Soest, Germany) mice were divided into four groups (time point T1: start of intervention): (i) a CD group; (ii) a CD plus vitamin E group (CD + E; 4.5 mg/kg body weight per day); (iii) a HFD group (high fat diet: 54 kJ % fat ssniff EF acc.D12492 (I) mod., ssniff Spezialdiäten GmbH, Soest, Germany); and (iv) a HFD plus vitamin E group (HFD + E; 4.5 mg/kg body weight per day).

The drinking water of animals was supplemented with vitamin E: “Aqua E” [33] containing 20 IU d-α-tocopherol, 15 mg other tocopherols, and 2 mg tocotrienols per mL. Aqua E has been used to guarantee an equivalent Vitamin E absorption: according to Papas et al. (2007), Aqua E showed a better bioavailability in malabsorbing patients compared to conventional based supplements [33]. Body weight and food intake were measured weekly, water/vitamin E uptake as an average per cage daily. Animals were sacrificed by cervical dislocation after 4 months (T4: end of intervention).

### 2.1. SCGE (Single Cell Gel Electrophoresis) Assay

DNA migration was studied in hepatocytes and colonocytes of mice in SCGE assay. These experiments are based on the measurement of DNA migration in an electric field [34]. Cells from livers and colons were collected according to the method developed by Sasaki et al., (2000) [35]. Briefly, 1.0 g liver tissue was homogenized by use of a Potter Elvehjem-type (B. Braun, Melsungen, Germany) at 400 rpm in 4.0 mL chilled homogenization buffer (pH 7.5). Subsequently, the homogenates were centrifuged (800 g, 10 min, 4 °C). Colon cells were isolated by scratching from the colon mucosa and kept on ice in 2.0 mL homogenization buffer. The nuclei were re-suspended in LMPA (low melting point agarose, 0.5%, Gibco, Paisley, UK) and transferred to slides which were pre-coated with NMPA (normal melting point agarose, 1.0%, Gibco, Paisley, UK).

The experiments were carried out according to international guidelines for SCGE experiments [36]. After lysis (pH 10.0) and electrophoresis (30 min, 300 mA, 25 V, at 4 °C, pH > 13), the gels were stained with ethidium bromide (20 µg/mL, Sigma-Aldrich, Vienna, Austria). Three slides were prepared per experimental time point and 50 cells were evaluated from each slide. Slides were examined under a fluorescence microscope (Nikon EFD-3, Tokyo, Japan) using 25-fold magnification. DNA migration was determined with a computer-aided comet assay image analysis system (Comet Assay IV, Perceptive Instruments, Bury St Edmunds, UK).

### 2.2. Gene Expression Analysis

Colon and liver samples were stored at −80 °C. RNA and DNA were isolated from liver and colon using the AllPrep DNA/RNA/miRNA Universal Kit (Qiagen, Hilden, Germany) according to manufacturer’s protocol. The concentration were measured respectively purity controlled with a Picodrop100 (Picodrop, Hinxton, UK). Complementary DNA (cDNA) was synthesized from 1 μg of total RNA by reverse transcription using RT^2^ First Strand Kit (Qiagen, Hilden, Germany). cDNA was analyzed in real-time PCR using qPCR Primer Assays (Qiagen, Hilden, Germany) and RT^2^ SYBR Green Mastermix (Qiagen, Hilden, Germany) according to protocol. PCR conditions were as follows: initial step of 95 °C for 10 min, followed by 40 cycles of 95 °C for 15 s and 60 °C for 1 min, ending with melting curve analysis (gradient melting of the products was performed at 0.5 °C/10 s from 65 °C to 95 °C). Each sample was analyzed in duplicate, with normalization to the housekeeping gene glycerinaldehyde-3-phosphate-dehydrogenase (GAPDH) as an internal control.

### 2.3. Methylation Analysis

Two micrograms of genomic DNA was bisulfite converted with EpiTect^®^ Fast Bisulfite Conversion kit (Qiagen, Hilden, Germany) and amplified by PCR using the PyroMark PCR Kit (Qiagen, Hilden, Germany) according to manufacturer’s instructions with primers for *Dnmt1* and *MLH1* designed by PyroMark Assay Design SW 2.0 Software (Table 1).

The PCR was carried out in a total reaction volume of 25.0 μL, containing 12.5 μL Pyromark 2× PCR master mix, 10 pmol (*Dnmt1*) or 7 pmol (*MLH1*) of each primer, 2.5 μL Coralload Concentrate 10× (Qiagen, Hilden, Germany), and 10.0 ng (*Dnmt1*) or 15.0 ng (*MLH1*) bisulfite converted DNA. Thermocycling started with initial denaturation at 95 °C for 15 min, followed by 45 cycles at 94 °C for 30 s, 55.5 °C for 45 s, 72 °C for 45 s and a final extension at 72 °C for 10 min. PCR product quality was examined with agarose gel-electrophoresis. Analysis of CpG methylation was performed with a Pyromark Q24 MDx (Qiagen, Hilden, Germany).

### 2.4. Statistical Analyses

In SCGE assays, statistical analyses were performed using GraphPad Prism 5.02 (GraphPad Software, La Jolla, CA, USA). The means and SD of percent DNA in the comet tails of the nuclei from the different treatment groups were calculated. Group means were compared using Student’s *t*-test based on the means of three slides.

All statistical analyses of gene expression and methylation analyses were performed using IBM SPSS Advanced Statistics 20.0 (SPSS, Standford, CA, USA). All data are shown mean ± SD.

ΔCT values of each gene were calculated by normalization to the housekeeping gene GAPDH (ΔCT = CT-Target − CT-GAPDH). The ΔΔCT value shows the difference between the two groups. The ΔCT value of the control group was deducted from the ΔCT value of the vitamin E group (ΔΔCT = ΔCT-Vitamin E − ΔCT-Control). Relative changes in gene expression between the intervention and control group are determined by the 2^−ΔΔCT^ equation (fold change = 2^−ΔΔCT^). The Kolmogorov–Smirnov-Test was used to test the normality of the data distribution. To examine significant relationships, Mann–Whitney-U Test was used. The interaction between DNA damage and mean methylation was tested by Spearman correlation test. For all difference-of-mean and correlation tests *p*-values ≤ 0.05 were considered as significant.

## 3. Results

### 3.1. Body Weight, Food Intake and Vitamin E Uptake

**Figure 1:**
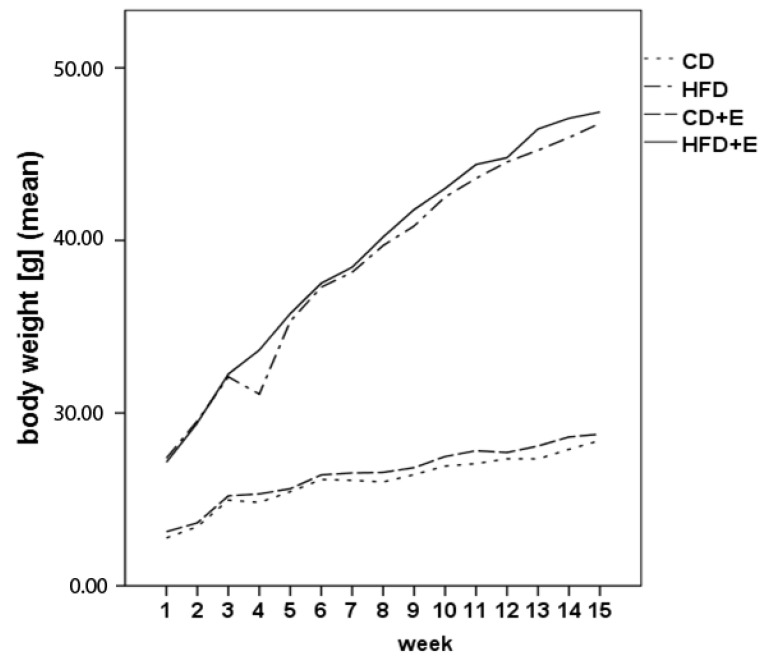
Body weight gain of C57BL/6J male mice over four months (*n* = 15) (CD = control diet; HFD = high fat diet; CD + E = control diet plus vitamin E; HFD + E = high fat diet plus vitamin E).

Body weight and food intake were measured weekly and water/vitamin E uptake as an average per cage daily. As shown in Table 2 food intake and total water consumption did not differ between the groups. The body weight of mice fed a HFD (T1: 32.57 ± 2.09 g; T4: 47.09 ± 0.83 g) and a HFD + E (T1: 32.77 ± 2.3 g; T4: 47.67 ± 0.49 g) increased significantly in comparison to CD-fed mice (T1: 24.66 ± 0.75 g; T4: 28.31 ± 0.24 g) and the CD + E group (T1: 24.94 ± 0.77 g; T4: 28.63 ± 0.14 g) over study period (*p* < 0.01, Figure 1). The body weight increase over study period was significant in all groups (*p* < 0.01, Figure 1).

Mean Vitamin E uptake was 3.90 ± 0.14 µL of Aqua E in the CD group and 5.89 ± 0.11 µL of Aqua E in the HFD group of each mouse per day. The α-tocopherol intake was 0.08 IU (CD) and 0.12 IU (HFD) per day and mouse.

### 3.2. SCGE Experiments from Colon and Liver Cells

**Figure 2:**
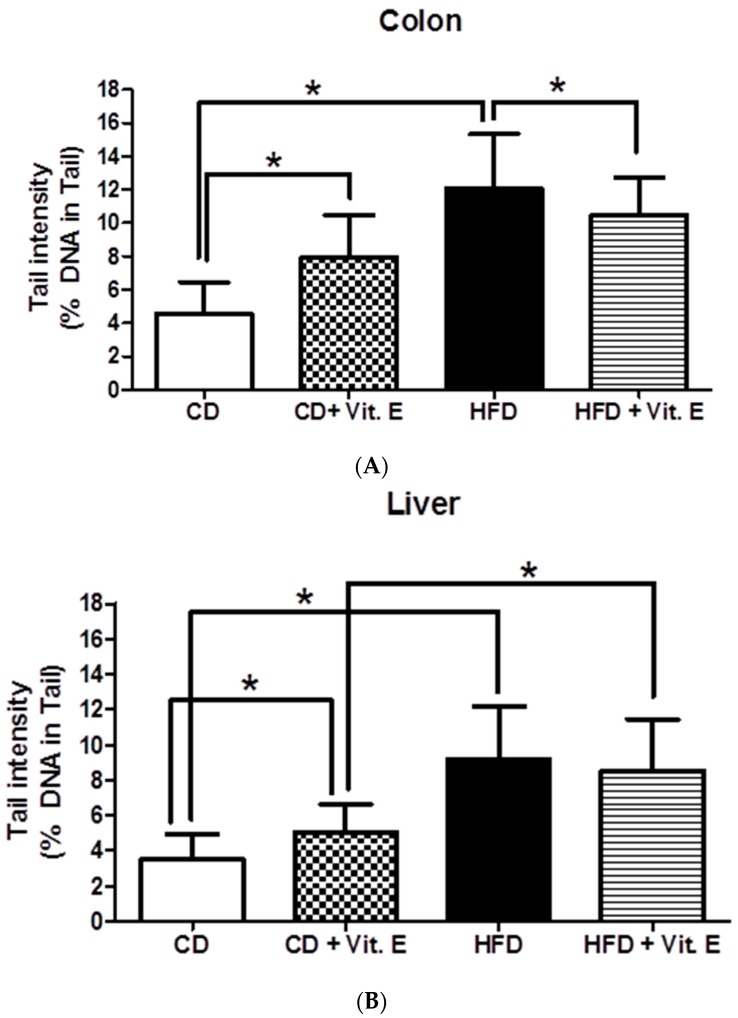
Impact of vitamin E supplementation on DNA damage in colon (**A**) and liver (**B**) of C57BL/6J male mice (*n* = 15). Bars indicate means ± SD of results obtained with 15 animals per group. From each sample, three slides were made and 50 cells were evaluated per slide. (CD = control diet; HFD = high fat diet; CD + E = control diet plus vitamin E; HFD + E = high fat diet plus vitamin E; * *p*-value ≤ 0.05).

HFD caused significant induction of DNA damage in both organs compared to CD (Figure 2). The extent of DNA migration was more pronounced in the colon (2.6-fold) than in the liver (2.3-fold). In HFD + E, the extent of DNA migration was significantly decreased by 17% (*p* ≤ 0.05) in the colon while no effect was seen in the liver compared to HFD fed mice (Figure 2). Supplementation with vitamin E in CD group caused significant DNA migration in both organs (1.7-fold in colon and 1.3-fold in liver).

### 3.3. Relative Gene Expression (Figure 3) and CpG Methylation (Figure 4) of Dnmt1 in Colon and Liver Cells

**Figure 3:**
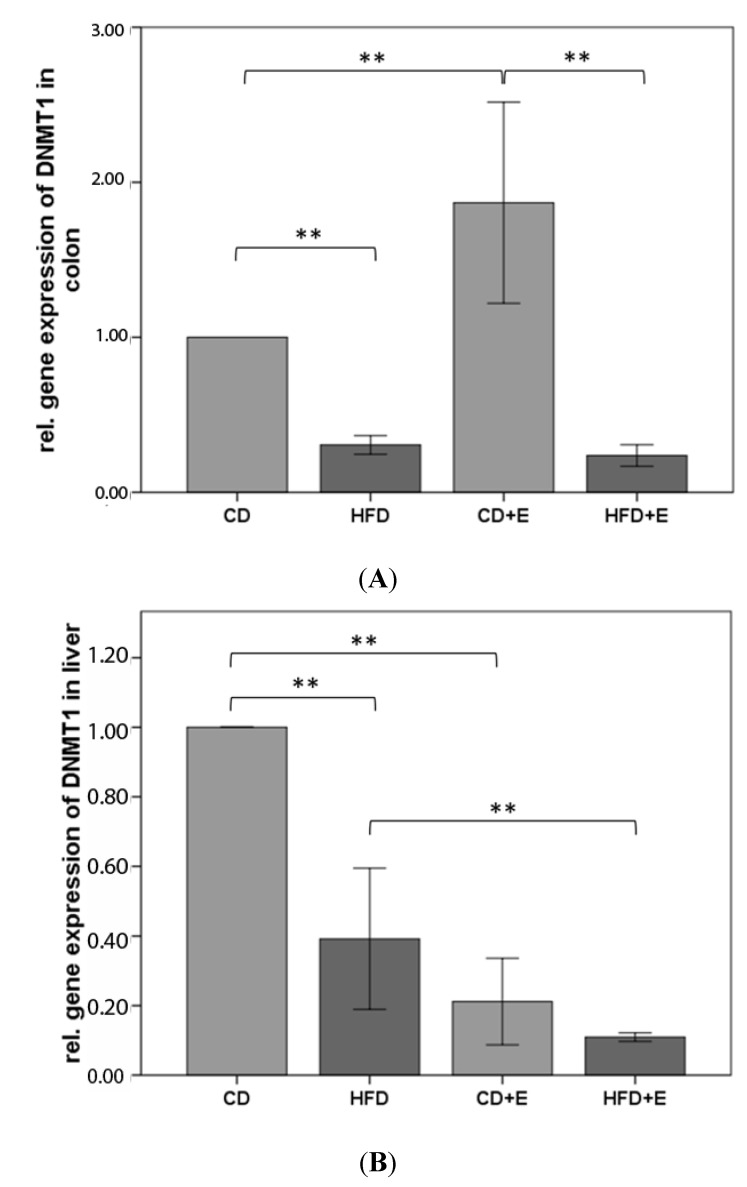
Relative gene expression of *Dnmt1* in colon (**A**) and liver (**B**) of C57BL/6J male mice (*n* = 15). Gene expression data were calculated relative to CD-data and normalized to the house keeping gene GAPDH. The error bar represents a 95% confidence interval. (CD = control diet; HFD = high fat diet; CD + E = control diet plus vitamin E; HFD + E = high fat diet plus vitamin E; ** *p*-value ≤ 0.01).

In colon cells, the relative expression of *Dnmt1* decreased for 61% in HFD compared to CD (*p* ≤ 0.01). With vitamin E supplementation *Dnmt1* relative gene expression was significantly lower (86%) in HFD + E in comparison to CD + E (*p* ≤ 0.01). The relative gene expression of *Dnmt1* in colon cells of HFD showed no significant differences in comparison to HFD + E (*p* = 0.394). Relative to CD mice, the vitamin E supplementation (CD + E) resulted in 87% higher expression of *Dnmt1* (*p* ≤ 0.01; Figure 3). In the liver, a significantly lower expression of *Dnmt1* in HFD compared to CD was shown with a reduction of 61% (*p* ≤ 0.01). CD compared to CD + E (79%) and HFD compared to HFD + E (68%) showed both a lower gene expression of *Dnmt1* in the liver (*p* ≤ 0.01). The relative gene expression of *Dnmt1* in liver cells was 25% lower in HFD + E compared to CD + E (*p* ≤ 0.01, Figure 3).

**Figure 4:**
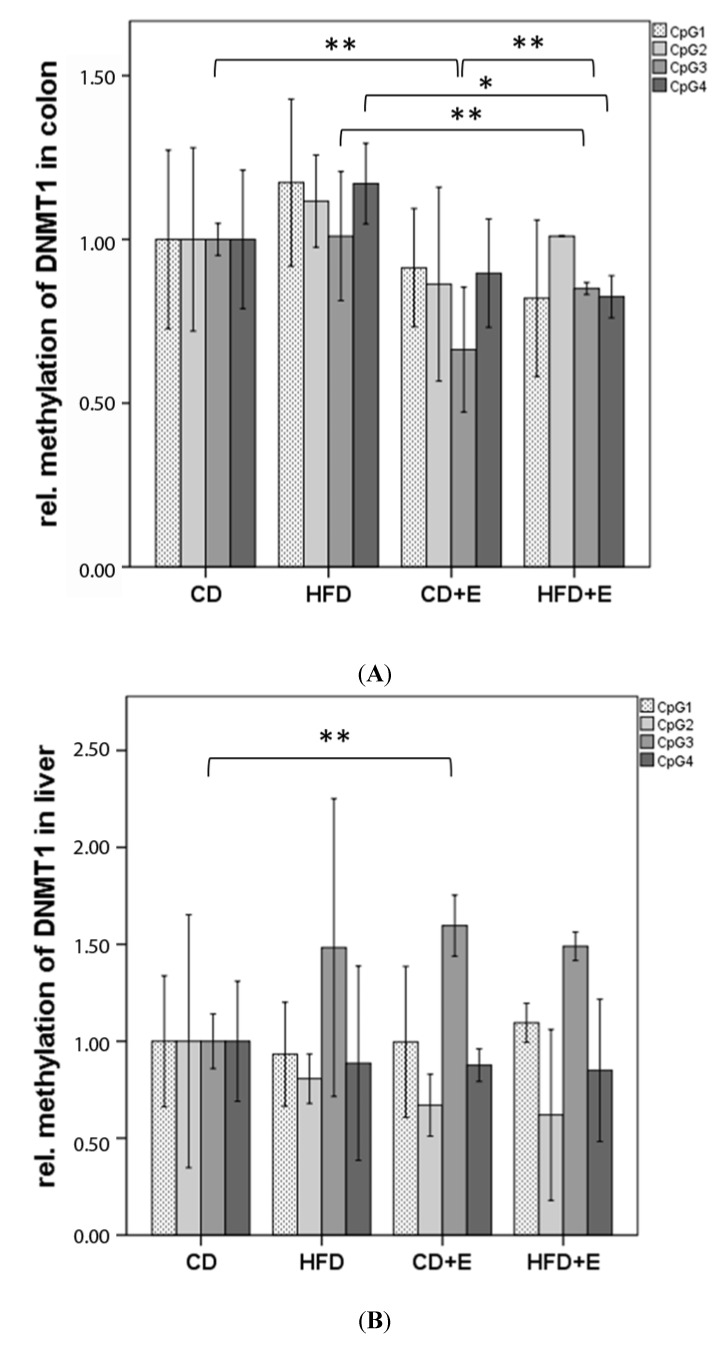
Relative CpG methylation status in promoter region of *Dnmt1* in colon (**A**) and liver (**B**) of C57BL/6J male mice (*n* = 15). All methylation data are relative to CD. The error bar represents a 95% confidence interval. (CD = control diet; HFD = high fat diet; CD + E = control diet plus vitamin E; HFD + E = high fat diet plus vitamin E; * *p*-value ≤ 0.05; ** *p*-value ≤ 0.01).

Four CpGs were analyzed in the promoter region of *Dnmt1* in liver and in colon (Figure 4). In colon cells, significant differences in methylation status were measured in CpG 3 and 4. In CD + E, CpG 3 showed 33.67% lower methylation (*p* ≤ 0.01) when compared to CD, and 25.67% lower when compared to HFD + E. Moreover, a significant decrease in methylation of both CpG 3 (17.13%, *p* ≤ 0.01) and CpG 4 (21.57%, *p* ≤ 0.05) was indicated in HFD + E in comparison to HFD. By comparing HFD with CD, a slight increase in methylation levels was observed in HFD, however, no significant difference was detected between those two groups (*p* = 0.394, Figure 4A). In CD a negative correlation between DNA damage and the mean methylation of *Dnmt1* in colon was seen (*r*^2^ = −0.837, *p* ≤ 0.05).

In liver cells, CpG 3 showed the highest relative methylation among all intervention groups. When compared to CD animals, increased methylation levels of CpG 3 were found in HFD, CD + E and HFD + E. Particularly in CD + E, vitamin E supplementation caused a significant hypermethylation of CpG 3 (59.67%, *p* ≤ 0.01). No significant difference in *Dnmt1* methylation in liver was observed between the HFD and HFD + E group (Figure 4B). Furthermore, in HFD DNA damage correlated positively with the mean methylation of *Dnmt1* in the liver (*r*^2^ = 0.956, *p* ≤ 0.01).

### 3.4. Relative Gene Expression (Figure 5) and CpG Methylation (Figure 6 and Figure 7) of MLH1 in Colon and Liver Cells

**Figure 5:**
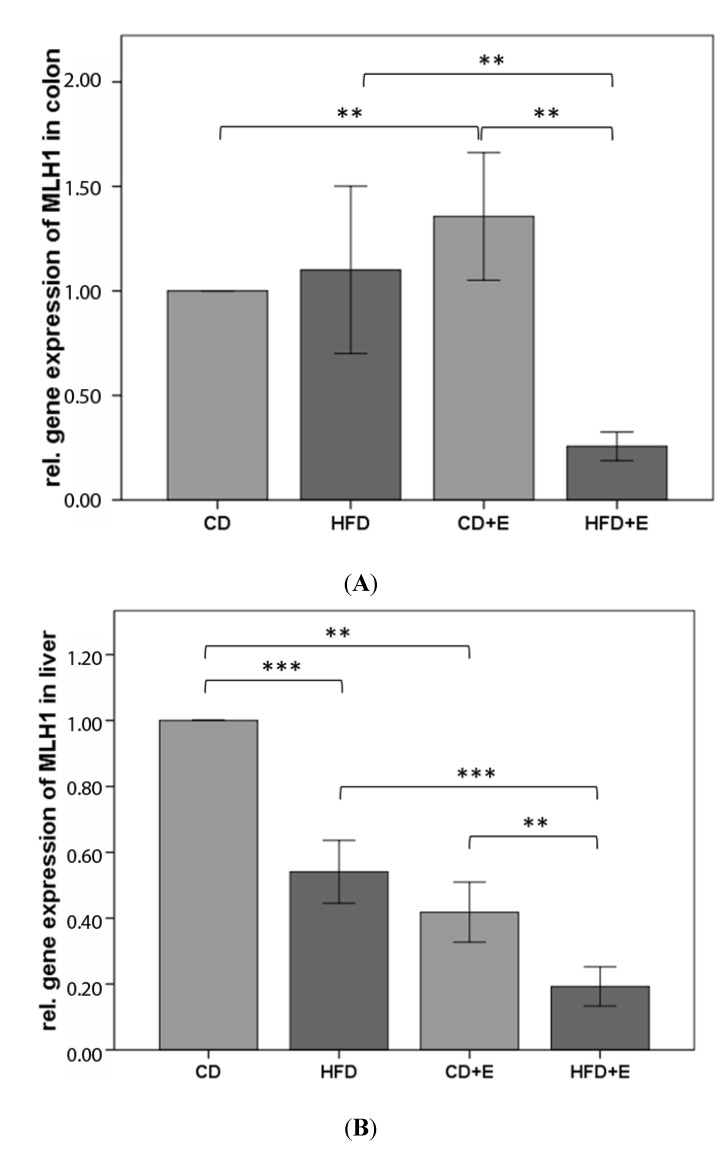
Relative gene expression of *MLH1* in colon (**A**) and liver (**B**) of C57BL/6J male mice (*n* = 15). Gene expression data were calculated relative to CD-data and normalized to the house keeping gene GAPDH. The error bar represents a 95% confidence interval. (CD = control diet; HFD = high fat diet; CD + E = control diet plus vitamin E; HFD + E = high fat diet plus vitamin E; ** *p*-value ≤ 0.01; *** *p*-value ≤ 0.001).

The relative gene expression of *MLH1* in colon did not result in significant differences between CD and HFD animals (*p* = 0.659). However, vitamin E supplementation induced a higher gene expression of *MLH1* in CD + E in comparison to CD (36%, *p* ≤ 0.01). In contrast vitamin E supplementation in HFD caused a significant reduction of 72% compared to HFD (*p* ≤ 0.01, Figure 5). The relative gene expression of *MLH1* in liver was significantly lower in the HFD animals (49%) compared to CD and HFD + E (53%) compared to HFD. Vitamin E supplementation induced a significant (58%) lower expression of *MLH1* in liver compared to CD (*p* ≤ 0.01; Figure 5).

**Figure 6:**
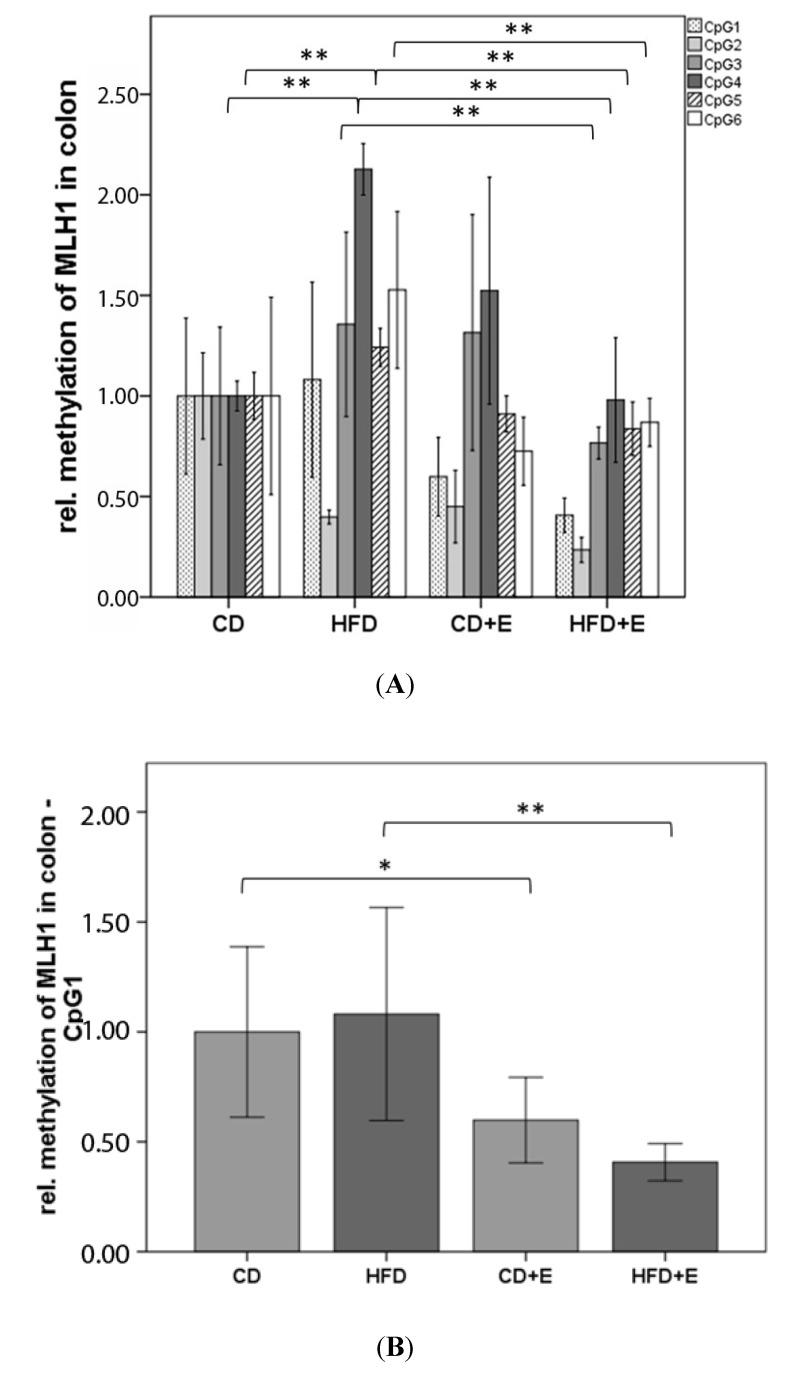

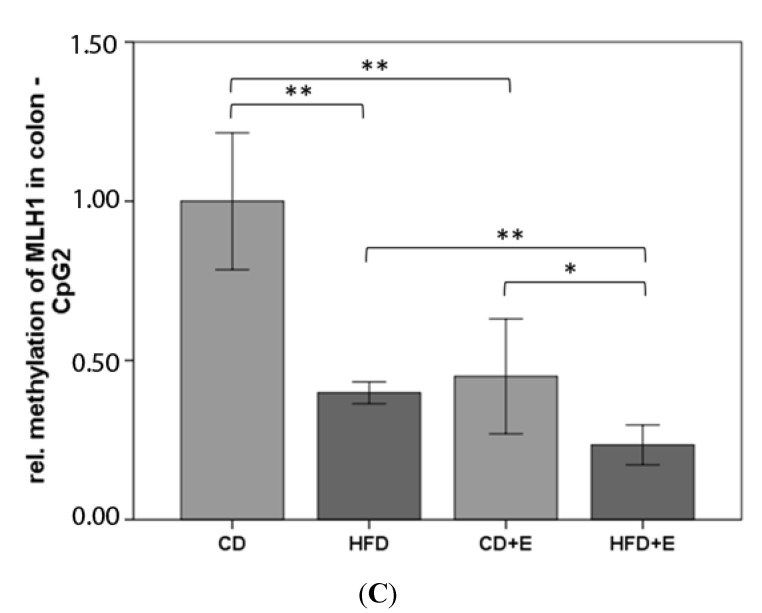
Relative CpG methylation status in promoter region of *MLH1* in colon of C57BL/6J male mice (*n* = 5): (**A**) mean methylation status for *MLH1* in colon is shown as an overview; (**B**) the methylation status of CpG 1; and (**C**) the methylation status of CpG 3 is specified. All methylation data are shown relative to CD. The error bar represents a 95% confidence interval. (CD = control diet; HFD = high fat diet; CD + E = control diet plus vitamin E; HFD + E = high fat diet plus vitamin E; * *p*-value ≤ 0.05; ** *p*-value ≤ 0.01).

**Figure 7:**
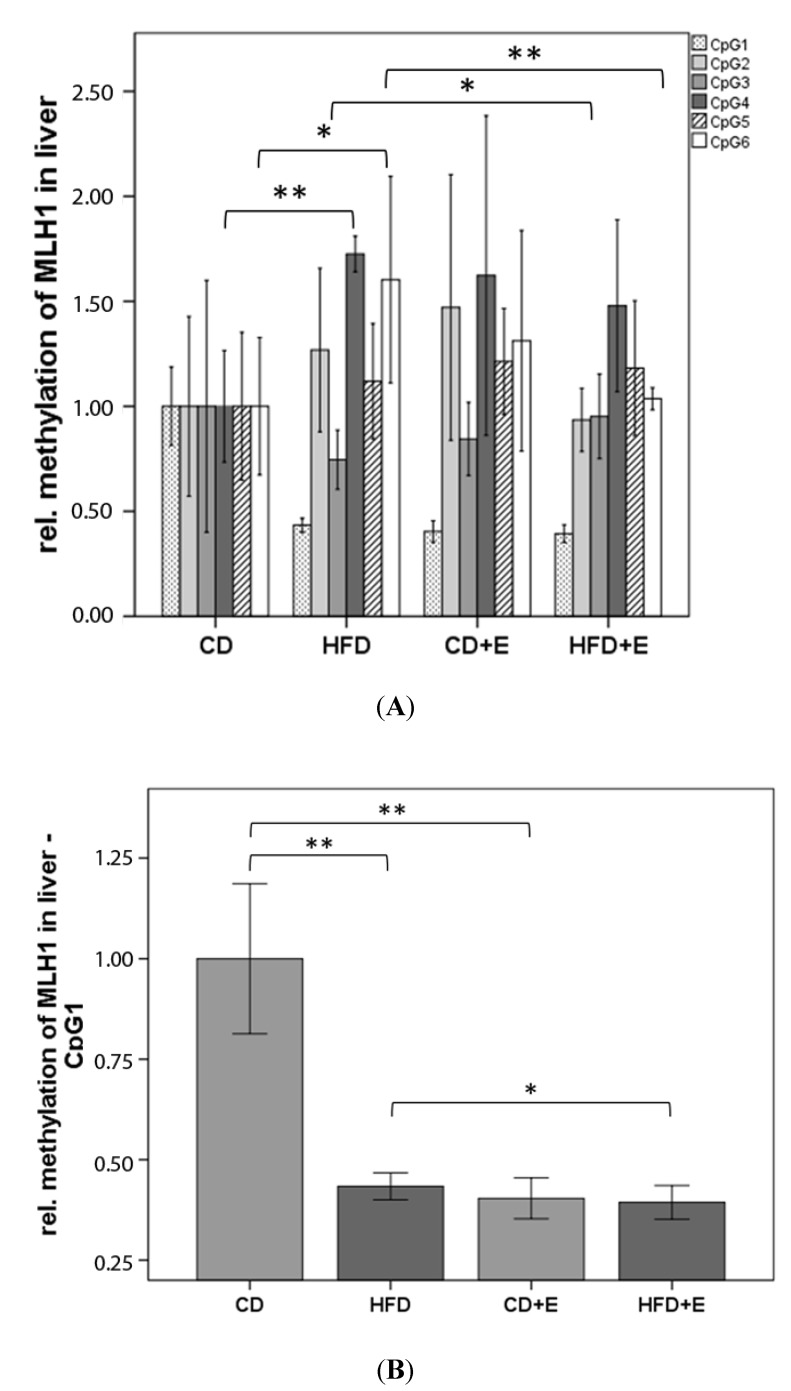
Relative CpG methylation status in promotor region of *MLH1* in liver (*n* = 15). Mean methylation data are shown for: *MLH1* in the liver as an overview (**A**) and CpG 1 (**B**). All methylation data are shown relative to CD. The error bar represents a 95% confidence interval. (CD = control diet, HFD= high fat diet, CD + E = control diet plus vitamin E, HFD + E = high fat diet plus vitamin E, * *p*-value ≤ 0.05, ** *p*-value ≤ 0.01).

In the *MLH1* promoter region, the relative methylation was analyzed for six CpGs in colon (Figure 6) and liver (Figure 7). In colon cells, vitamin E supplementation significantly reduced methylation of CpG 1 (CD + E: 40.17%, *p* ≤ 0.05; HFD + E: 59.30%, *p* ≤ 0.01) and 2 (CD + E: 55.00%, *p* ≤ 0.01; HFD + E: 76.53%, *p* ≤ 0.01) in comparison to CD (Figure 6A,B). The same effect has been observed over all six CpGs in HFD + E compared to HFD (all *p* ≤ 0.01). Significantly different methylation levels between CD and HFD were found in CpG 2, 4 and 5 (all *p* ≤ 0.01). In general, HFD showed a higher relative methylation over all CpGs with exception of CpG 2, where *MLH1* methylation decreased by 60.17% in comparison to CD (*p* ≤ 0.01) (Figure 6A,C). In liver cells, similar to the colon, hypomethylation of CpG 1 was found in both supplementation groups (CD + E: 59.63%; HFD + E: 60.63%; all *p* ≤ 0.01) in comparison to CD, and the methylation of CpG 1 was reduced by 56.63% in HFD (*p* ≤ 0.01) (Figure 7B). On the contrary, the HFD showed significant hypermethylation of CpG 4 (72.11%, *p* ≤ 0.01) and CpG 6 (11.93%, *p* ≤ 0.05) when compared to CD. In comparison to HFD, vitamin E treatment (HFD + E) significantly decreased the methylation levels of CpG 1 (9.23%, *p* ≤ 0.05) and 6 (35.37%, *p* ≤ 0.01), whereas methylation of CpG 3 significantly increased by 27.57% (*p* ≤ 0.05) (Figure 7A).

## 4. Discussion

HFD induced a significant increase of DNA damage in liver and colon compared with CD in mice. *Dnmt1* relative gene expression decreased significantly in both organs, whereas methylation status showed a slight increase in HFD compared to CD. The relative gene expression of *MLH1* in colon did not show significant differences between the two different diets, while, in liver, significantly lower *MLH1* expression was shown in HFD compared to CD. The methylation status of all CpGs was generally higher in HFD in comparison to CD.

Although IL-6 expression was below the detection limit in our experiment, low-grade inflammation is known as a major cause of obesity caused by the release of FFAs from adipocytes, related to the increased amount of adipose tissue. FFAs are usually stored as triglycerides or provide energy through β-oxidation by cell’s mitochondria. Minor products, ROS, are potentially harmful for cellular functions. A complex antioxidant system provides protection, although an imbalance due to obesity results in oxidative stress, potentiating comorbidities. Antioxidant therapies have been shown to reduce the oxidative stress, reduce susceptibility of low-density lipoprotein (LDL) to oxidation, inhibit secretion of pro-inflammatory cytokines [37], improve insulin signaling in vitro [38], and improve glycemic control in individuals with type 2 diabetes [39,40,41]. In vivo, vitamin E no longer improves insulin sensitivity [42], only transient improvements were shown [41,43].

Vitamin E interrupts lipid peroxidation due to the presence of the phenolic hydroxyl group on the chroman ring of the molecule resulting in tocopheroxyl radicals, which are regenerated by means of hydrogen donors. However, due to incomplete reduction, tocopheroxyl radicals can also induce oxidative stress by reaction with polyunsaturated fatty acids in the LDL particles. Thus, Aqua E has been used in concentrations in accordance to recommended daily allowance, and further studies testing different concentrations would be of interest.

Natural vitamin E comprises four tocopherols and four tocotrienols. Therefore, we used a mixture of tocopherols and tocotrienols, which reflects the human diet more accurately than pure α- tocopherol, used in previous animal and human studies. All forms of the vitamin E family are absorbed and delivered to the liver. Only α-tocopherol accumulates in this organ, whereas the other isoforms are rapidly metabolized and excreted. The accumulation of mainly α-tocopherol in hepatic tissue is the consequence of the expression of a cytosolic protein (α-tocopherol transfer protein, α-TTP) with high selectivity for α-tocopherol and low or very low affinity for the other tocopherols [44]. α-TTP and other bound vitamin E forms are prevented from being catabolized in the liver. Among the isoforms γ-tocopherol is slightly less efficient than α-tocopherol as a scavenger of oxygen radicals, but it is an efficient scavenger of reactive nitrogen species [45]. In addition, tocotrienols have more pronounced cancer protective effects than tocopherol [46], and tocotrienols are notably also more effective in NF-κB inhibition than tocopherols [47].

### 4.1. Vitamin E Protects DNA Damage Caused by HFD

We found a significant increase of DNA damage with HFD in both organs. Vitamin E supplementation decreased DNA damage. Bardowell et al (2012) estimated that unmetabolized tocopherols and tocotrienols are discarded via biliary excretion in feces [48]. This observation may provide an explanation for more pronounced protective effects in the colon. Ju et al. [49] evaluated 32 animal studies published since 1980 with regard to cancer-preventive activities of tocopherols and tocotrienols. Only 12 studies focused on colon tumorigenesis and aberrant crypt foci formation, and only two out of twelve studies showed a protective effect of vitamin E family compounds in colon. Vitamin E has also been shown to protect against liver damage induced by oxidative stress in animal experiments [50,51].

Taken together, this study showed that supplementation of Aqua E with HFD for four months significantly improves DNA damage in colon and liver of mice. This identifies vitamin E as an important nutritional factor in the prevention of DNA damage caused by oxidative stress due to obesity although the dosage has to be taken into account.

### 4.2. Vitamin E Supplementation Affects Specific CpG Sites of Dnmt1, Resulting in Altered Relative Gene Expression of Dnmt1

As mentioned above, oxidative stress due to obesity is involved in both genome-wide hypomethylation and promoter hypermethylation of the DNA [8] as oxygen radicals impair DNA lesions. These lesions interfere with methylation activity since damaged DNA cannot serve as acceptor for methyl groups, causing global hypomethylation [9,10].

The methyl donor SAM and the expression of *Dnmt1* are also impaired by oxidative stress [17,18]. Both are important in the maintenance of epigenetic modifications by adding methyl groups to the C5 position of cytosine in CpG dinucleotides at the replication fork and are responsible to copy DNA methylation patterns to newly synthesized daughter strands [18]. Thus, altered expression of *Dnmt1* can lead to hypo- or hypermethylation of other genes, resulting in expression changes of mRNAs or proteins. Altered levels of *Dnmt1* can disrupt cellular mechanisms and may lead to pathological changes of different gene functions [18].

We showed a lower expression of *Dnmt1* in colon of HFD with no changes due to vitamin E supplementation, although in CD animals an increase with supplementation was noted (Figure 3). Methylation status of CpG 3 and 4 in the promoter region of *Dnmt1* in colon is significantly lower in CD + E compared to CD and HFD + E. The same CpGs showed a significantly lower methylation in HFD + E compared with HFD control group. HFD showed a slightly higher promoter methylation status compared to CD (Figure 4). In all intervention groups, a decreased gene expression was noted in liver (Figure 3) although the methylation status of CpG 3 was increased in HFD, CD + E as well as in HFD + E. However, no significant differences were observed between the two HFD groups, while in the CD + E group vitamin E supplementation caused a significant hypermethylation of CpG 3 (Figure 4). Furthermore, in the liver, a positive correlation of *Dnmt1* mean methylation and DNA damage has been observed in liver whereas in CD a correlation has been found in the colon.

Given those observations, it may be concluded that vitamin E induces tissue specific changes in *Dnmt1* gene expression and relative promoter methylation, which presumably depend on the host metabolic state (lean vs. obese). In addition to this statement, different results in colon and liver may be explained by vitamin E metabolism. It is known that all vitamin E isomers undergo intestinal absorption and afterwards are taken up by liver. However, only α-tocopherol can be retained in hepatocytes, which is primarily due to the hepatic α-TTP that preferentially binds to α-tocopherol and prevents its hepatic catabolism [52]. Other forms, in contrary, are fast metabolized and excreted, with about 80% of the total metabolites being eliminated via fecal route [52]. γ-tocopherol and tocotrienols are thought to have anti-inflammatory and anti-oxidative properties not shared by α-form [53]. Furthermore, particular metabolites that may accumulate in colon have been shown to inhibit pro-inflammatory pathways more strongly than unmetabolized vitamin E forms [52].

In addition, some mechanistic studies suggested that vitamin E exerts its anti-oxidative and anti-inflammatory activities by modulating transcriptional factors via *Dnmt1*-dependent route. One of them is NF-κB, a pro-inflammatory factor activated by ROS [54]. It is reported that NF-κB stimulates *Dnmt1* through cytokine-dependent pathway, and vice versa, inhibition of NF-κB led to reduction in *Dnmt1* expression [55]. Tocotrienols and especially α- tocopheryl succinate have been recognized as effective inhibitors of NF-κB [56], and all of them were supplied by the vitamin E supplement used in our study. However, we did not detect a higher gene expression of *IL-6* between the groups in colon and liver. Upritchard et al. (2012) showed a significant decrease of the inflammatory status in type 2 diabetics due to vitamin E supplementation (800 IU/day), indicated by decreased plasma levels of C reactive protein (CRP) [57]. Inhibition of IL-1β release decreases the expression of IL-6 and further of CRP [37]. However, an antioxidant-independent effect via a decrease of 5-lipoxygenase activity is suggested [58], although no changes of inflammatory markers and of plasma CRP levels were observed due to vitamin E supplementation (800 IU/day and 1200 IU/day) in overweight individuals [41]. Adverse effects were also shown, such as increased risk for heart failure [59] or increased risk of hemorrhagic stroke [60]. Furthermore, tumor suppressor genes or cell cycle regulation may be affected leading to aberrant cell growth [20]. Thus, a routine vitamin E supplementation due to obesity is not recommended at the present.

Recent research indicated the requirement of an ubiquitin interacting motif (UIM) in the N-terminal regulatory domain of *Dnmt1*, which binds to ubiquitinated H3 tails and is essential for DNA methylation in vivo. H3 ubiquitination and subsequent DNA methylation were shown to require UHRF1 (Ubiquitin-like, Containing PHD and RING Finger Domains, 1) PHD (plant homeodomain) binding to H3R2 [61]. In addition, we should consider that the methylation status and gene expression are only snapshots, and cell cycle information is missing. However, Fuks et al. (2000) disclosed the interaction of *Dnmt1* with histone deacetylase activity and repression of gene transcription in vivo [62]. In mice with diet induced obesity (DIO), the binding of HDACs is increased at the leptin promoter whereas histones H3 and H4 are hypoacetylated, lysine 4 of histone H3 (H3K4) is hypomethylated. The methylation and the binding of DNMTs and methyl-CpG-binding domain protein 2 (MBD2) are increased and RNA Pol II is decreased, resulting in a negative correlation of leptin promoter methylation and leptin gene transcription. These modifications may indicate a feedback loop for the maintenance of leptin concentrations due to obesity [63]. In another DIO mouse model, *Dnmt1* expression and enzymatic activity were elevated in adipocytes, leading to promoter hypermethylation and following decreased adiponectin expression [55].

Potential compensatory effects in response to a lack or oversupply of methyl groups for DNA methylation may also affect *Dnmt1* expression [64]. The sequence [5′-TTTCCGCG-3′] within the genomic methylation analysis (CpG 1 and 2 in our study), was identified as crucial site for the transcriptional regulation of *Dnmt1* by the transcription factor E2F1 [65,66]. However, we were not able to show significant changes on these specific CpG sites. These results disclose the various mechanisms controlling *Dnmt1* activity and the multifaceted interplay between DNA and histone modifications, or even the diverse effects on other CpG methylation of target gene promoters. Thus, we were not able to elucidate how and if *Dnmt1* regulates the MMR system in the current study, although a coherence is shown with *MLH1*.

### 4.3. Vitamin E Supplementation Affects Specific CpG Sites of MLH1, Inducing a Lower Gene Expression of MLH1 with High-Fat Diet

*MLH1* is part of the MMR system that is responsible for ensuring overall DNA integrity [14]. Enhanced oxidative stress, as a consequence of overweight and obesity, can cause elevated DNA damage [67] which in turn requires optimal function of the MMR system, including *MLH1*.

We showed a higher gene expression of *MLH1* in CD + E in comparison to CD and HFD in colon, whereas in HFD + E *MLH1* gene expression decreased (Figure 5). The methylation status of six CpGs in the promoter region of *MLH1* in colon showed a higher methylation over all s in comparison to CD (Figure 6). In addition, vitamin E supplementation induced a lower methylation of specific CpG sites in both supplemented groups, also shown in liver cells. Vitamin E supplementation induced a lower expression of *MLH1* in liver (Figure 5). Both HFD groups also had a lower expression compared to CD. CpG 4 and 6 were significantly hypermethylated in HFD compared to CD. Comparisons between HFD and HFD + E showed significant differences in methylation levels of three CpG sites (Figure 7).

Switzeny et al. (2012) showed a significant higher CpG methylation in two particular *MLH1* promoter regions. The methylation status and DNA strand breaks correlated significantly, although no changes in gene expression of *MLH1* due to dietary intervention with folate in non-insulin dependent diabetes mellitus type 2 was shown [24]. Thus, our results are in accordance with previously published data using antioxidants. Sinicrope et al. (2015) indicated a “less likely” deficient MMR in colon cancers from obese patients, suggesting that obesity-associated colon cancers are predominantly caused by sufficient MMR, a molecular subtype showing chromosomal instability with significantly worse survival rates. However, only the deficient MMR colon cancers are associated with a higher DNA methylation near gene promoter regions of *MLH1*. Higher estradiol levels in both sexes due to obesity might be the cause of the lower frequency of deficient MMR system [68].

In summary, differences in gene expression might indicate tissue specificity, different metabolic pathways, especially as a higher nutrient bioavailability is indicated in colon, main transit organ, but not in the metabolizing organ (liver), with substance dependence in the enterohepatic pathway or the transport system (blood). Differences in methylation status might already indicate an adaption to dietary intervention, although the duration is not sufficient for ROS-dependent defects in gene expression. Differences of methylation status of different CpG-sites in promoter regions are rarely known, although some CpG sites show more profound results between our groups. The involvement of other epigenetic modifications has to be taken also into account: DNA methylation at gene promoters regulates gene expression through a complicated mechanism involving multiple modifications, including histone modifications and miRNAs. Similar results seen in in vitro experiments with Caco2 cells will be submitted for publication soon.

## 5. Conclusions

Our study with C57BL/6J male mice fed a HFD or CD with or without supplemental vitamin E shows significant effects of HFD on DNA damage, analyzed in SCGE assays. HFD also resulted in significant organ-specific changes in the epigenetically important *Dnmt1* gene and the DNA repair gene *MLH1*. Vitamin E reduced DNA damage and affected *Dnmt1* and *MLH1* gene expression and methylation, which was also organ specific. These results suggest that intervention with vitamin E, as an epigenetic active food ingredient, can be developed as an effective prevention of obesity-related and oxidative stress-induced health risks.

## Figures and Tables

**Table 1:** Sequence to analyze and primers for CpG methylation analysis.

Gene	Primer	Sequence (5′→3′)	Size (bp)	GC%
*DNMT1*	FW	Biotin - GTA GGT TGT AGA AGA TAG AAT AGT TTT GA	29	31
	RW	CCC ACT CTC TTA CCC TAT ATA ATA CAT	27	37
	Seq	CCC CTC CCA ATT AAT TTC	18	44.4
	Sequence ID: gb|AH009208.2| *DNMT1*: at reverse strand of chromosome 9: 20907205–20959888 (52684 bp).			
Sequence to analyze	7104-CGCGCGCGCGAAAAAGCCGGGGTCTCGT-7131		27	7 CpGs
*MLH1*	FW	AGG GTA TTT TAG TTT TTA TTG GTT GGA GA	29	31
	RW	TTA CAC CTC AAT TCC TAA AAT CTC TAT CCC – Biotin	30	37
	Seq	TTT AGT TTT TAG AAA TGA GTT AAT A	25	16
	Sequence ID: ref|XR_379849.3 *MLH1*: at reverse strand of Chromosome 9: 111228228–111271786 (43559 bp)			
Sequence to analyze	19-GAAGAGCGGACCGTGAACTTTGACGCGCAAGCGCG TTGCCTTCTA-GCCTGGTGTCGGGCCGCTG-82		64	8 CpGs

**Table 2:** Body weight, food and water intake of C57BL/6J male mice over a period of four months.

		Chow Intake (g)				Water Intake (mL)				Weight (g)			
Month		1	2	3	4	1	2	3	4	1	2	3	4
**Intervention**	**CD**	2.64 ± 0.07	2.11 ± 0.01	2.08 ± 0.04	2.06 ± 0.03	5.58 ± 0.21	5.29 ± 0.22	5.39 ± 0.28	4.95 ± 0.56	24.66 ± 0.75	26.17 ± 0.16	27.17 ± 0.18	28.31 ± 0.24
	**CD + E**	2.70 ± 0.08	2.70 ± 0.05	2.76 ± 0.06	2.76 ± 0.51	5.80 ± 0.21	5.76 ± 0.15	5.97 ± 0.17	5.55 ± 0.27	24.94 ± 0.77	26.58 ± 0.15	27.75 ± 0.19	28.63 ± 0.14
	**HFD**	2.56 ± 0.04	2.59 ± 0.02	2.60 ± 0.02	2.56 ± 0.06	5.34 ± 0.20	4.93 ± 0.24	5.10 ± 0.14	5.01 ± 0.18	32.57 ± 2.09	39.00 ± 1.37	43.97 ± 1.02	47.09 ± 0.83
	**HFD + E**	2.51 ± 0.02	2.45 ± 0.06	2.50 ± 0.05	2.54 ± 0.01	5.21 ± 0.20	4.69 ± 0.06	5.05 ± 0.04	5.09 ± 0.11	32.77 ± 2.3	39.49 ± 1.64	44.67 ± 1.23	47.67 ± 0.49

## Abbreviations

α-TTP	α-tocopherol transfer protein
CAT	catalase
CD	control diet
CD + E	control diet plus vitamin E
cDNA	complementary DNA
CRP	C reactive protein
CYPs	cytochrome P450s
DIO	diet induced obesity
*Dnmt1*	DNA methyltransferase 1
FFAs	free fatty acids
GAPDH	glycerinaldehyd-3-phosphat-Dehydrogenase
GPX	glutathione peroxidase
H	histone
HFD	high fat diet
HFD + E	high fat diet plus vitamin E
IL-6	interleukin-6
LDL	low-density lipoprotein
LMPA	low melting point agarose
MBD2	methyl-CpG-binding domain protein 2
MCP-1	monocyte chemoattractant protein-1
MGMT	O6-methylguanine-deoxyribonucleic acidmethyltransferase
*MLH1*	MutL homolog 1
MMR	DNA mismatch repair
MSI	microsatellite instability
NADPH	nicotinamide adenine dinucleotide phosphate
NAFLD	non-alcoholic fatty liver diseases
NMPA	normal melting point agarose
Nrf2	Nuclear factor-erythroid 2-related factor 2
PAI-1	plasminogen activator inhibitor-1
PHD	plant homeodomain
ROS	reactive oxygen species
SAM	S-Adenosylmethionin
SCGE	single cell gel electrophoresis
SOD	superoxide dismutase
TNFα	tumor necrosis factor α
T	time point
UHRF1	Ubiquitin-like, Containing PHD and RING Finger Domains, 1
UIM	ubiquitin interacting motif

## References

[1] WHO Consultation on Obesity (2000). Obesity: Preventing and managing the global epidemic. Report of a WHO consultation. World Health Organ. Tech. Rep. Ser..

[2] Fazel S., Luigi G., Daglia M., Mohammad S. (2015). Role of quercetin as an alternative for obesity treatment: You are what you eat!. Food Chem..

[3] Fernández-Sánchez A., Madrigal-Santillán E., Bautista M., Esquivel-Soto J., Morales-González Á., Esquivel-Chirino C., Durante-Montiel I., Sánchez-Rivera G., Valadez-Vega C., Morales-González J.A. (2011). Inflammation, oxidative stress, and obesity. Int. J. Mol. Sci..

[4] Kershaw E.E., Flier J.S. (2004). Adipose Tissue as an Endocrine Organ. J. Clin. Endocrinol. Metab..

[5] Savini I., Catani M.V., Evangelista D., Gasperi V., Avigliano L. (2013). Obesity-associated oxidative stress: Strategies finalized to improve redox state. Int. J. Mol. Sci..

[6] Le Lay S., Simard G., Martinez M.C., Andriantsitohaina R. (2014). Oxidative Stress and Metabolic Pathologies: From an Adipocentric Point of View. Oxid. Med. Cell. Longev..

[7] Furukawa S., Fujita T., Shimabukuro M., Iwaki M., Yamada Y., Nakajima Y., Nakayama O., Makishima M., Matsuda M., Shimomura I. (2004). Increased oxidative stress in obesity and its impact on metabolic syndrome. J. Clin. Investig..

[8] Thanan R., Oikawa S., Hiraku Y., Ohnishi S., Ma N., Pinlaor S., Yongvanit P., Kawanishi S., Murata M. (2014). Oxidative Stress and Its Significant Roles in Neurodegenerative Diseases and Cancer. Int. J. Mol. Sci..

[9] Franco R., Schoneveld O., Georgakilas A.G., Panayiotidis M.I. (2008). Oxidative stress, DNA methylation and carcinogenesis. Cancer Lett..

[10] Donkena K.V., Young C.Y.F., Tindall D.J. (2010). Oxidative stress and DNA methylation in prostate cancer. Obstet. Gynecol. Int..

[11] Ziech D., Franco R., Pappa A., Panayiotidis M.I. (2011). Reactive oxygen species (ROS)--Induced genetic and epigenetic alterations in human carcinogenesis. Mutat. Res..

[12] Chahal M., Xu Y., Lesniak D., Graham K., Famulski K., Christensen J.G., Aghi M., Jacques A., Murray D., Sabri S. (2010). MGMT modulates glioblastoma angiogenesis and response to the tyrosine kinase inhibitor sunitinib. Neuro Oncol..

[13] Kovalchuk O., Burke P., Besplug J., Slovack M., Filkowski J., Pogribny I. (2004). Methylation changes in muscle and liver tissues of male and female mice exposed to acute and chronic low-dose X-ray-irradiation. Mutat. Res..

[14] Harfe B.D., Jinks-Robertson S. (2000). DNA mismatch repair and genetic instability. Annu. Rev. Genet..

[15] Peltomäki P. (2003). Role of DNA mismatch repair defects in the pathogenesis of human cancer. J. Clin. Oncol..

[16] Li G.-M. (2008). Mechanisms and functions of DNA mismatch repair. Cell Res..

[17] Hitchler M.J., Domann F.E. (2007). An epigenetic perspective on the free radical theory of development. Free Radic. Biol. Med..

[18] Subramaniam D., Thombre R., Dhar A., Anant S. (2014). DNA methyltransferases: A novel target for prevention and therapy. Front. Oncol..

[19] Verma M., Chattopadhyay B.D., Paul B.N. (2013). Epigenetic regulation of DNMT1 gene in mouse model of asthma disease. Mol. Biol. Rep..

[20] Hodge D.R., Xiao W., Clausen P.A., Heidecker G., Szyf M., Farrar W.L. (2001). Interleukin-6 regulation of the human DNA methyltransferase (HDNMT) gene in human erythroleukemia cells. J. Biol. Chem..

[21] Hodge D.R. (2005). Interleukin 6 Supports the Maintenance of p53 Tumor Suppressor Gene Promoter Methylation. Cancer Res..

[22] Hodge D.R., Cho E., Copeland T.D., Guszczynski T., Yang E., Seth A.K., Farrar W.L. (2007). IL-6 enhances the nuclear translocation of DNA cytosine-5-methyltransferase 1 (DNMT1) via phosphorylation of the nuclear localization sequence by the AKT kinase. Cancer Genom. Proteom..

[23] Remely M., Ferk F., Sterneder S., Setavesh T., Roth S., Kepcija T., Noorizadeh R., Rebhan I., Greunz M., Beckmann J. (2017). EGCG prevents high-fat diet-induced changes in gut microbiota, decreases of DNA strand breaks, and changes in expression and DNA methylation of Dnmt1 and MLH1 in C57BL/6J male mice. Oxid. Med. Cell. Longev..

[24] Switzeny O.J., Mullner E., Wagner K.H., Brath H., Aumuller E., Haslberger A.G. (2012). Vitamin and antioxidant rich diet increases MLH1 promoter DNA methylation in DMT2 subjects. Clin. Epigenetics.

[25] Colombo M.L. (2010). An update on vitamin E, tocopherol and tocotrienol-perspectives. Molecules.

[26] Mocchegiani E., Costarelli L., Giacconi R., Malavolta M., Basso A., Piacenza F., Ostan R., Cevenini E., Gonos E.S., Franceschi C. (2014). Vitamin E-gene interactions in aging and inflammatory age-related diseases: Implications for treatment. A systematic review. Ageing Res. Rev..

[27] Stone W.L., Papas A.M. (1997). Tocopherols and the etiology of colon cancer. J. Natl. Cancer Inst..

[28] Traussnigg S., Kienbacher C., Halilbasic E., Rechling C., Kazemi-Shirazi L., Hofer H., Munda P., Trauner M. (2015). Challenges and Management of Liver Cirrhosis: Practical Issues in the Therapy of Patients with Cirrhosis due to NAFLD and NASH. Dig. Dis..

[29] Dias F.M., Leffa D.D., Daumann F., de Marques S.O., Luciano T.F., Possato J.C., de Santana A.A., Neves R.X., Rosa J.C., Oyama L.M. (2014). Acerola (Malpighia emarginata DC.) juice intake protects against alterations to proteins involved in inflammatory and lipolysis pathways in the adipose tissue of obese mice fed a cafeteria diet. Lipids Health Dis..

[30] Hakkak R., Korourian S., Pavliv O., Evans T., Melnyk S. (2014). Effects of obesity on development of oxidative stress and DNA damages in liver of the obese Zucker rat (643.1). FASEB J..

[31] Al-Aubaidy H.A., Jelinek H.F. (2011). Oxidative DNA damage and obesity in type 2 diabetes mellitus. Eur. J. Endocrinol..

[32] Collins A.R., Oscoz A.A., Brunborg G., Gaivão I., Giovannelli L., Kruszewski M., Smith C.C., Stetina R. (2008). The comet assay: Topical issues. Mutagenesis.

[33] Papas K., Kalbfleisch J., Mohon R. (2007). Bioavailability of a novel, water-soluble vitamin E formulation in malabsorbing patients. Dig. Dis. Sci..

[34] Tice R.R., Agurell E., Anderson D., Burlinson B., Hartmann A., Kobayashi H., Miyamae Y., Rojas E., Ryu J.C., Sasaki Y.F. (2000). Single cell gel/comet assay: Guidelines for in vitro and in vivo genetic toxicology testing. Environ. Mol. Mutagen..

[35] Sasaki Y.F., Kawaguchi S., Kamaya A., Ohshita M., Kabasawa K., Iwama K., Taniguchi K., Tsuda S. (2002). The comet assay with 8 mouse organs: Results with 39 currently used food additives. Mutat. Res..

[36] Burlinson B., Tice R.R., Speit G., Agurell E., Brendler-Schwaab S.Y., Collins A.R., Escobar P., Honma M., Kumaravel T.S., Nakajima M. (2007). Fourth International Workgroup on Genotoxicity testing: Results of the in vivo Comet assay workgroup. Mutat. Res..

[37] Devaraj S., Jialal I. (1998). The effects of alpha-tocopherol on critical cells in atherogenesis. Curr. Opin. Lipidol..

[38] Maddux B.A., See W., Lawrence J.C., Goldfine A.L., Goldfine I.D., Evans J.L. (2001). Protection Against Oxidative Stress—Induced Insulin Resistance in Rat L6 Muscle Cells by Micromolar Concentrations of -Lipoic Acid. Diabetes.

[39] Paolisso G., D’Amore A., Galzerano D., Balbi V., Giugliano D., Varricchio M., D’Onofrio F. (1993). Daily vitamin E supplements improve metabolic control but not insulin secretion in elderly type II diabetic patients. Diabetes Care.

[40] Jacob S., Ruus P., Hermann R., Tritschler H., Maerker E., Renn W., Augustin H., Dietze G., Rett K. (1999). Oral administration of rac-α-lipoic acid modulates insulin sensitivity in patients with type-2 diabetes mellitus: A placebo-controlled pilot trial. Free Radic. Biol. Med..

[41] Manning P.J., Sutherland W.H.F., Walker R.J., Williams S.M., De Jong S.A., Ryalls A.R., Berry E.A. (2004). Effect of high-dose vitamin E on insulin resistance and associated parameters in overweight subjects. Diabetes Care.

[42] Picklo M.J., Thyfault J.P. (2015). Vitamin E and vitamin C do not reduce insulin sensitivity but inhibit mitochondrial protein expression in exercising obese rats. Appl. Physiol. Nutr. Metab..

[43] Barbagallo M., Dominguez L.J., Tagliamonte M.R., Resnick L.M., Paolisso G. (1999). Effects of Vitamin E and Glutathione on Glucose Metabolism. Hypertension.

[44] Sato Y., Hagiwara K., Arai H., Inoue K. (1991). Purification and characterization of the alpha-tocopherol transfer protein from rat liver. FEBS Lett..

[45] Raederstorff D., Wyss A., Calder P.C., Weber P., Eggersdorfer M. (2015). Vitamin E function and requirements in relation to PUFA. Br. J. Nutr..

[46] Krebs und Ernährung - Thieme.de - Thieme Webshop - Siegfried Knasmüller. https://www.thieme.de/shop/Endokrinologie--Diabetologie/Knasmueller-Krebs-und-Ernaehrung-9783131542113/p/000000000269440101.

[47] Aggarwal B.B., Sundaram C., Prasad S., Kannappan R. (2010). Tocotrienols, the vitamin E of the 21st century: Its potential against cancer and other chronic diseases. Biochem. Pharmacol..

[48] Bardowell S.A., Duan F., Manor D., Swanson J.E., Parker R.S. (2012). Disruption of mouse cytochrome p450 4f14 (Cyp4f14 gene) causes severe perturbations in vitamin E metabolism. J. Biol. Chem..

[49] Ju J., Picinich S.C., Yang Z., Zhao Y., Suh N., Kong A.-N., Yang C.S. (2010). Cancer-preventive activities of tocopherols and tocotrienols. Carcinogenesis.

[50] Factor V.M., Laskowska D., Jensen M.R., Woitach J.T., Popescu N.C., Thorgeirsson S.S. (2000). Vitamin E reduces chromosomal damage and inhibits hepatic tumor formation in a transgenic mouse model. Proc. Natl. Acad. Sci. USA.

[51] Kakizaki S., Takagi H., Fukusato T., Toyoda M., Horiguchi N., Sato K., Takayama H., Nagamine T., Mori M. (2001). Effect of alpha-tocopherol on hepatocarcinogenesis in transforming growth factor-alpha (TGF-alpha) transgenic mice treated with diethylnitrosamine. Int. J. Vitam. Nutr. Res..

[52] Jiang Q. (2014). Natural forms of vitamin E: Metabolism, antioxidant, and anti-inflammatory activities and their role in disease prevention and therapy. Free Radic. Biol. Med..

[53] Jiang Q., Ames B.N. (2003). Gamma-Tocopherol, but not alpha-tocopherol, decreases proinflammatory eicosanoids and inflammation damage in rats. FASEB J..

[54] Glauert H.P. (2007). Vitamin E and NF-κB Activation: A Review. Vitam. Horm..

[55] Kim A.Y., Park Y.J., Pan X., Shin K.C., Kwak S.-H., Bassas A.F., Sallam R.M., Park K.S., Alfadda A.A., Xu A. (2015). Obesity-induced DNA hypermethylation of the adiponectin gene mediates insulin resistance. Nat. Commun..

[56] Nakamura T., Goto M., Matsumoto A., Tanaka I. (1998). Inhibition of NF-kappa B transcriptional activity by alpha-tocopheryl succinate. Biofactors.

[57] Upritchard J.E., Sutherland W.H., Mann J.I. (2000). Effect of supplementation with tomato juice, vitamin E, and vitamin C on LDL oxidation and products of inflammatory activity in type 2 diabetes. Diabetes Care.

[58] Devaraj S., Jialal I. (1999). Alpha-tocopherol decreases interleukin-1 beta release from activated human monocytes by inhibition of 5-lipoxygenase. Arterioscler. Thromb. Vasc. Biol..

[59] Lonn E., Bosch J., Yusuf S., Sheridan P., Pogue J., Arnold J.M.O., Ross C., Arnold A., Sleight P., Probstfield J. (2005). Effects of long-term vitamin E supplementation on cardiovascular events and cancer: A randomized controlled trial. JAMA.

[60] Sesso H.D., Buring J.E., Christen W.G., Kurth T., Belanger C., MacFadyen J., Bubes V., Manson J.E., Glynn R.J., Gaziano J.M. (2008). Vitamins E and C in the prevention of cardiovascular disease in men: The Physicians’ Health Study II randomized controlled trial. JAMA.

[61] Qin W., Wolf P., Liu N., Link S., Smets M., La Mastra F., Forné I., Pichler G., Hörl D., Fellinger K. (2015). DNA methylation requires a DNMT1 ubiquitin interacting motif (UIM) and histone ubiquitination. Cell Res..

[62] Fuks F., Burgers W.A., Brehm A., Hughes-Davies L., Kouzarides T. (2000). DNA methyltransferase Dnmt1 associates with histone deacetylase activity. Nat. Genet..

[63] Shen W., Wang C., Xia L., Fan C., Dong H., Deckelbaum R.J., Qi K. (2014). Epigenetic modification of the leptin promoter in diet-induced obese mice and the effects of N-3 polyunsaturated fatty acids. Sci. Rep..

[64] Martínez J.A., Milagro F.I., Claycombe K.J., Schalinske K.L. (2014). Epigenetics in adipose tissue, obesity, weight loss, and diabetes. Adv. Nutr..

[65] Kimura H., Nakamura T., Ogawa T., Tanaka S., Shiota K. (2003). Transcription of mouse DNA methyltransferase 1 (Dnmt1) is regulated by both E2F-Rb-HDAC-dependent and -independent pathways. Nucleic Acids Res..

[66] McCabe M.T., Davis J.N., Day M.L. (2005). Regulation of DNA Methyltransferase 1 by the pRb/E2F1 Pathway. Cancer Res..

[67] Roberts C.K., Sindhu K.K. (2009). Oxidative stress and metabolic syndrome. Life Sci..

[68] Sinicrope F.A., Foster N.R., Yoon H.H., Smyrk T.C., Kim G.P., Allegra C.J., Yothers G., Nikcevich D.A., Sargent D.J. (2012). Association of obesity with DNA mismatch repair status and clinical outcome in patients with stage II or III colon carcinoma participating in NCCTG and NSABP adjuvant chemotherapy trials. J. Clin. Oncol..

